# Knowledge and Implementation of Tertiary Institutions’ Social Health Insurance Programme (TISHIP) in Nigeria: a case study of Nnamdi Azikiwe University, Awka

**DOI:** 10.11604/pamj.2017.28.171.11379

**Published:** 2017-10-24

**Authors:** Maureen Ugonwa Anetoh, Chiamaka Henrietta Jibuaku, Sunday Odunke Nduka, Samuel Uchenna Uzodinma

**Affiliations:** 1 Department of Clinical Pharmacy and Pharmacy Management, Nnamdi Azikiwe University, Awka, Anambra State, Nigeria

**Keywords:** Students attitudes, Patient satisfaction, health promotion, Professional practice

## Abstract

**Introduction:**

Tertiary Institutions’ Social Health Insurance Programme (TISHIP) is an arm of the National Health Insurance Scheme (NHIS), which provides quality healthcare to students in Nigerian higher institutions. The success of this scheme depends on the students’ knowledge and awareness of its existence as well as the level of its implementation by healthcare providers. This study was therefore designed to assess students’ knowledge and attitude towards TISHIP and its implementation level among health workers in Nnamdi Azikiwe University Medical Centre.

**Methods:**

Using a stratified random sampling technique, 420 undergraduate students of Nnamdi Azikiwe University, Awka were assessed on their level of awareness and general assessment of TISHIP through an adapted and validated questionnaire instrument. The level of implementation of the scheme was then assessed among 50 randomly selected staff of the University Medical Center. Data collected were analyzed using Statistical Package for Social Sciences (SPSS) version 20 software.

**Results:**

Whereas the students in general, showed a high level of TISHIP awareness, more than half of them (56.3%) have never benefited from the scheme with 52.8% showing dissatisfaction with the quality of care offered with the scheme. However, an overwhelming number of the students (87.9%) opined that the scheme should continue. On the other hand, the University Medical Centre staff responses showed a satisfactory scheme implementation.

**Conclusion:**

The study found satisfactory TISHIP awareness with poor attitude among Nnamdi Azikiwe University students. Furthermore, the University Medical Centre health workers showed a strong commitment to the objectives of the scheme.

## Introduction

In order to provide effective and efficient health care for their citizens especially, the poor and vulnerable [1], most developing countries recently initiated the implementation of some health financing strategies focusing mainly on, social health insurance scheme. These reform programmes and strategies were aimed at providing easy access to healthcare at an affordable price through various prepayment systems with expected results of improving the overall health status of the citizens. Nigeria, like most other African countries, keyed into this Health Insurance Scheme programme by enacting the National Health Insurance Scheme Act, 1999 with the aim of providing easy access to qualitative, equitable and affordable healthcare for all Nigerians [2]. The first phase of this scheme was rolled out in 2005, with the formal sector social health insurance programme being on the lead [3,4]. This formal sector social health insurance scheme recognizes the principal beneficiary and the spouse with four biological children below the age of 18 years, with more dependents only recognized on additional contributions.

As a means of catering for the healthcare needs of Nigerians who may not have been captured by the formal sector scheme, more schemes including the Tertiary Institutions’ Social Health Insurance Programme (TISHIP) were introduced. The TISHIP is a social security system whereby the health care of students in tertiary institutions in Nigeria is paid for from funds pooled through compulsory contributions involving the students and the government. The programme promotes the health of students with a view to creating conducive learning environment and uninterrupted academic activities due to poor health [5]. It applies to students in universities, colleges of education, polytechnics, schools of nursing and midwifery and other specialized colleges including monotechnics without any discrimination or segregation [6]. This programme was therefore established to ensure access to qualitative service for students in tertiary institutions thus promoting the health of the students with a view to creating conducive learning environment.

The knowledge of TISHIP, its acceptance, and utilization by the students as well as, its proper implementation in the health care facilities is crucial for the actualization of its goals and general benefits. Previous studies revealed that students barely visit institutions’ health care facilities due to high costs of medical services, poor working conditions and inadequate referral services [6] most of which TISHIP is meant to address. It has also been shown that most Nigerian policies such as this, are often poorly understood with a resultant low level of knowledge about its standard procedures and regulations [7]. This study, therefore, was designed to assess students’ awareness and attitude to TISHIP as well as to assess the level of implementation of the scheme among Nnamdi Azikiwe University Medical Centre healthcare workers.

## Methods

### Study design and sampling

This study was a cross-sectional prospective study carried out amongst Nnamdi Azikiwe University students and the staff of the University Medical Centre sampled from four campuses of the University located at Awka, Nnewi, Agulu and Mbaukwu between February and July, 2015. A desired sample size of 396 students calculated from a total number of 43, 396 students of the University (Department of Students’ Affairs records) using Yamane sample size calculation technique was considered ideal for the study. However, to account for potential refusals and incorrect filling of some questionnaires, an additional percentage of the participating students were added bringing the total number of students to 420. The 420 students were sampled using a stratified random sampling technique. Then, 50 health workers comprising of the medical doctors, pharmacists, medical laboratory scientists, nurses and others in the medical centre were randomly selected and used for the study.

### Survey instrument

Two research questionnaires were used for the study. The first questionnaire, adapted from previous studies [1, 8, 9] with validation was used for the students’ assessment. This instrument consisted of three sections that assessed demographic characteristics, TISHIP awareness and general assessment of TISHIP by the students. The second questionnaire used to assess the extent of implementation of the scheme by the health workers was developed using the objectives of NHIS and independently face validated by four pharmacy lecturers and an NHIS staff in Anambra state. A pilot study was then carried out on both instruments to ensure the internal consistency of the instruments, using 30 students and 10 staff who did not participate in the main study. Results of the pilot study were used to further modify the questionnaires to improve on clarity and flow. The questionnaires were then, distributed to respondents and retrieved after filling.

### Data analysis

The data collected from the questionnaires were entered into an excel sheet and analyzed using a computer Software Package for Social Sciences version 20.0. Descriptive statistics was calculated for the demographic variables. Quantitative data were analyzed by computing frequency tables, means, proportions (percentages) and descriptive cross tabulations. Categorical variables were summarized using frequencies and percentages.

### Ethical considerations

Full ethical approval with document number, NAUTH/CS/66/Vol.7/120 was obtained from the Ethical Review Board of Nnamdi Azikiwe University Teaching Hospital, Nnewi. Oral consent was obtained from the participants.

## Results

**Figure 1:**
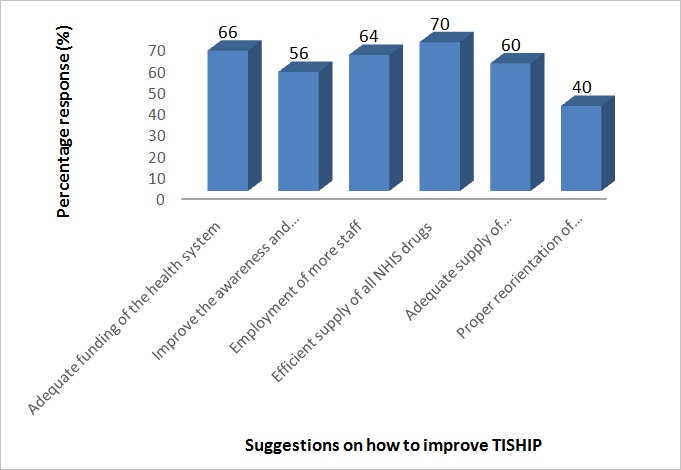
Percentage response on how to improve TISHIP suggested by health workers

A total number of 470 questionnaires were distributed (420 for students and 50 for health care workers) with 94.3% and 100% retrieval rate for the students and healthcare workers respectively. The demographic characteristics of the students (Table 1) indicated that more than 90 % of the students were single and between 16 and 27 years of age. The surveyed health workers showed more staff above 45 years (22 %) with almost equal distributions among the other age ranges. Doctors, Pharmacists and Nurses made up 66 % of the workers with the nurses having the greatest number of participants (36 %). On the students’ knowledge and awareness of this scheme as summarized in Table 2, majority of the students accessed healthcare outside the school clinic (52.2%) and only about one-third (37.6 %) reported accessing healthcare in the school clinic. Although all the students reported to have known about TISHIP through various means, only about two-third got the actual meaning of TISHIP. Quite a good number of the students agreed that TISHIP promotes healthcare delivery in Nigeria (Strongly agree (27.6 %); Agree (60.9 %)) and also, ensures that every student in the higher institution has access to quality healthcare (Strongly agree (22.9%); Agree (60.9%)). A summary of the students’ assessment of TISHIP program (Table 3) showed that only about half of the students believed that the institution is utilizing this program with about one-third not actually sure of its utilization although, more than half of the respondents had benefited from the scheme. The healthcare professionals’ attitude was reported to be unfriendly by 28.8% of the students with 52.8 % not feeling satisfied with the quality of care they received. However, an overwhelming number of the students (87.9%) were of the opinion that TISHIP should continue. The major drawbacks to TISHIP implementation as reported by the students (Table 4) was lack of essential drugs in addition to others like long waiting time and poor service delivery (35.9%), poor attitude and behavior of service providers (30.6 %) and inadequate knowledge and awareness of the scheme (26.8 %). Health workers responses to TISHIP implementation (Table 5) indicated sound knowledge and an overall good satisfaction with TISHIP with an increased students’ visit to the clinic since the onset of this programme. However, more than two-third of the staff (70.0%) reported poor availability of the TISHIP drugs in the clinic without reimbursement when drugs are sourced from outside the clinic. Among the points suggested by the health workers on how to improve TISHIP as presented in Figure 1, efficient supply of drugs (70%), adequate funding of the health system (66%) and enrollment of more staff (64%) ranked high.

**Table 1:** Socio-demographic characteristics of the respondents

	Students		Healthcare Workers	
Variable		Response n (%)		Response n (%)
**Age**				
	16-21	188 (47.5)	20-25	5 (10.0)
	22-27	184 (46.5)	26-30	9 (18.0)
	28-32	14 (3.5)	31-35	9 (18.0)
	33-38	7 (1.8)	36-40	8 (16.0)
	39-43	1 (0.3)	41-45	8 (16.0)
	44-49	2 (0.5)	>45	11 (22.0)
**Sex**				
	Male	203 (51.3)	Male	15 (30.0)
	Female	193 (48.7)	Female	35 (70.0)
**Marital Status**				
	Single	368 (92.9)	Single	11 (22.0)
	Married	28 (7.1)	Married	38 (76.0)
			Widow/Widower	1 (2.0)
**Classification**				
	Pre- degree/ Pre-science	24 (6.1)	Doctor	6 ( 12.0)
	Diploma	52 (13.1)	Pharmacist	9 (18.0)
	Regular	297 (75.0)	Med Lab scientist	5 (10.0)
	Continuous Education Programme (CEP)	7 (1.8)	Nurse	18 (36.0)
	Post graduate	16 (4.0)	Radiographer	2 (4.0)
			Others	10 (20.0)

**Table 2:** Responses to TISHIP knowledge and awareness by the students

Variable	Response n (%)			
**Where the students access healthcare**				
School Clinic	149 (37.6)			
Hospital outside the school	96 (24.2)			
Pharmacy outside the school	87 (22.0)			
Patent medicine vendors	24 (6.1)			
Never been sick	40 (10.1)			
**Meaning of TISHIP**				
Tertiary Institution Social Health Insurance Programme	250 (63.1)			
Today’s Institution Students Health Insurance Programme	6 (1.5)			
Today’s Institutions Social Health Insurance Programme	8 (2.0)			
Tertiary Insurance Students Health Institutions Programme	12 (3.0)			
**How they got to know about TISHIP**				
Friends	152 (38.4)			
During Orientation	81 (20.5)			
While at the school clinic	95 (24.0)			
Information media Don’t Know TISHIP	68 (17.2) 0 (0)			
	**SA**	**A**	**D**	**SD**
**TISHIP promotes improvement in health care delivery in Nigeria**	109 (27.6)	241 (60.9)	38 (9.6)	8 (2.0)
**TISHIP ensures that every student in higher institution has access to quality healthcare**	91 (22.9)	241 (60.9)	51(12.9)	13(3.3)
**TISHIP ensures that parents and guardians are protected from the financial hardship of huge medical bills**	103 (26.0)	198 (50.0)	70 (17.7)	25 (6.3)
**Both students’ and government contributions are involved in TISHIP**	75 (18.9)	231 (58.3)	77 (19.4)	13 (3.3)

TISHIP= Tertiary Institution Social Health Insurance Programme

SA=Strongly Agree, A= Agree, D= Strongly Disagree, SD= Strongly Disagree

**Table 3:** Students’ assessment of TISHIP Program

Variable	Responsen (%)
**Scheme utilization by the institution**	
Yes	226 (57.1)
No	34 (8.6)
I don’t Know	136 (34.3)
**Ever benefited from the scheme**	
Yes	223 (56.3)
No	173 (43.7)
**Ever paid money at the clinic under TISHIP**	
Yes	40 (10.1)
No	356 (89.9)
**The attitude of the healthcare professionals**	
Aggressive	46 (11.6)
Friendly	132 (33.3)
Professional	150 (37.9)
Indifferent	68 (17.2)
**Received all prescribed medications**	
Yes	194 (49.0)
No	202 (51.0)
**Referral in special cases**	
Yes	92 (23.2)
No	304 (76.8)
**Payment in the referred hospital**	
Yes	44 (11.1)
No	48 (12.1)
Not applicable	304 (76.8)
**Satisfaction with the quality of care offered by TISHIP**	
Yes	187 (47.2)
No	209 (52.8)
**Discontinuation of TISHIP**	
Yes	48 (12.1)
No	348 (87.9)

**Table 4:** Draw backs to TISHIP reported by the students

Factor	Percentage Response
Insufficient knowledge and awareness of the scheme	26.8
Health care providers deny enrollees full entitlement	13.4
Some providers charge additional fees	9.8
Poor attitude and behavior of service providers	30.6
Long waiting time and poor service delivery	35.9
Shortage/lack of essential drugs	78.3
Poor level of cleanliness in the clinic (Non-conducive environment)	11.9
Lack of privacy and inadequate visiting hours	13.6

**Table 5:** Responses by health workers on implementation of TISHIP

Implementation Question	Responsen (%)		
	**Yes**	**No**	**I don’t Know**
Knowledge of the TISHIP scheme	46 (92.0)	4 (8.0)	n/a
The school clinic makes use of TISHIP scheme	46 (92.0)	4 (8.0)	n/a
Improvement in the availability of working materials for providers since the onset of the programme	34 (68.0)	16 (32.0)	n/a
The students’ visit to the clinic increased with the onset of the programme	44 (88.0)	6 ( 12.0)	n/a
All the NHIS listed drugs are available in the school clinic	15 ( 30.0)	35 (70.0)	n/a
All the prescribed drugs are always provided to the patients	23 (46.0)	24 (48.0)	3 ( 6.0)
There is reimbursement when the patients buy from retail pharmacy	3 (6.0)	33 (66.0)	14 (28.0)
Students are being referred to NHIS accredited hospitals for specialist attention	47 (94.0)	3 (6.0)	n/a
Students are attended to promptly in the referred NHIS centres	32 (64.0)	2 (4.0)	16 (32.0)
Satisfaction with the TISHIP scheme	38 (76.0)	12 (24.0)	n/a
n/a=Not applicable			

## Discussion

The result of this study showed a high level of TISHIP knowledge and awareness with poor attitude, however, among Nnamdi Azikiwe University students as well as a satisfactory scheme implementation report by the healthcare workers in the institution’s Medical Centre. Although this study has some limitations like the use of one hospital and small sample size, the result indicated the need for proper and wider assessment of TISHIP activities in Nigerian higher institutions to ensure that the objectives of this programme are maximized. National Health Insurance Scheme was enacted in 1999 in Nigeria to ensure the provision of standard quality of healthcare which is a fundamental human right [10, 11]. One of the several programmes included in this scheme is the Tertiary Institutions Social Health Insurance Programme (TISHIP) designed to provide health insurance to students in tertiary institutions. Knowledge and level of awareness of health insurance including satisfaction level were identified as critical issues to the implementation of NHIS in Nigeria [2].

This study found a high level of knowledge and awareness of the TISHIP programme among the surveyed students as indicated by the large number of respondents that agreed that TISHP is designed to promote healthcare delivery in Nigeria while ensuring access to quality healthcare with the involvement of both the students and government in TISHIP contributions. This good knowledge and awareness was shown to be gained through various means including association with friends, during orientation and then, through social media among others. However, the fact that only about half of the students were aware of TISHIP utilization by their medical center is indicative of some problems with the programme implementation in the institution. This problem may likely be due to lack of proper orientation which is where the students are expected to receive first-hand information about the objectives and benefits of the scheme. This problem in orientation may also be seen from the small number of the students that got to know about TISHIP through orientation means. This percentage that believed on the utilization of the programme by the institution may have influenced the number of students that have benefited from it. About 89.9% of the students who have been treated did not pay for treatment. This is in line with the set objective that the students are treated and given most of their drugs without payment [5]. However, not all medications/drugs are captured in the NHIS list implying that the drugs not captured in the list will be paid for. Therefore, the few that indicated paying under the scheme could be for drugs or other services which were not included in the NHIS accredited drugs or services list [5].

Contrary to other findings [1,12], our study found that more than half of the students showed dissatisfaction with TISHIP quality of care but, the overwhelming number that opined that TISHIP should not be discontinued is indicative of their belief in the programme. Some of the drawbacks to TISHIP as reported by the students which may have affected the overall satisfaction include lack of essential drugs, poor attitude and staff behavior including, long waiting time and poor service delivery among others. Most of these drawbacks are in line with those mentioned by other researchers even in the parent programme [8, 13, 14]. On the other hand, the University Medical Centre staff showed a positive outlook on the scheme’s implementation. They also reported an increased inflow of students to the clinic since the onset of the programme. However, a good number of the health workers reported non-availability of NHIS drugs in the clinic which was in agreement with the students’ report. Furthermore, the staff reported that students are not paid back when they purchase their drugs elsewhere contrary to NHIS operational guideline, 2012 [5]. These reflect some of the bottleneck suffered by most government policies and programmes in developing countries. Although the majority of the health workers reported being satisfied with TISHIP, most of them suggested NHIS drugs supply, adequate funding of the health system, enrollment of more health personnel and adequate supply of medical equipment and infrastructure as ways of improving TISHIP implementation to help maximize its objectives. Other suggested ways of improving TISHIP implementation include improved awareness and proper reorientation of both the staff and students. Most of these factors have been suggested by other researchers [9, 15, 16] as ways of improving NHIS in Nigeria.

## Conclusion

This study found that the health workers in the study settings are really committed to the objectives of TISHIP with a positive outlook on the scheme. Although the students showed good knowledge and awareness of the programme, they however, are dissatisfied with some of the operations of the scheme but desired that the scheme be continued while all the lapses which include majorly shortage/lack of drugs, payment for some drugs and non-reimbursement after the purchase of some drugs be addressed.

### What is known about this topic

- The Federal Government of Nigeria rolled out its first phase of National Health Insurance scheme in 2005 and later introduced more schemes including the Tertiary Institutions’ Social Health Insurance Programme (TISHIP);
- Years after the initiation of NHIS in Nigeria, there is still low coverage rate and slow progress of health insurance which have been blamed on poor knowledge and poor client-provider relationship;
- Other important factors that have been identified to affect the utilization of NHIS and in particular, TISHIP include inadequate referral services, lack of essential drugs and dissatisfaction with services provided.

### What this study adds

- In line with the need for periodic assessment of new programmes with the purpose of making suggestions for improvements, this study observed a high TISHIP knowledge and awareness with poor attitude, however, among Nnamdi Azikiwe University students;
- A satisfactory scheme implementation report by the healthcare workers in the institution’s Medical Centre was further observed in the study;
- Surveyed students showed dissatisfaction with some of the scheme’s operations but desired that the scheme be continued while all the lapses which include majorly shortage/lack of drugs, payment for some drugs and non-reimbursement after the purchase of some drugs be addressed.

## Competing interests

The authors declare no competing interests.
